# Effects of Cardiorespiratory Fitness and Obesity on Salivary Secretory IgA and Alpha-Amylase in South African Children

**DOI:** 10.3390/children3030012

**Published:** 2016-07-30

**Authors:** Dorota E. Starzak, Kristen F. Konkol, Andrew J. McKune

**Affiliations:** 1 Discipline of Biokinetics, Exercise and Leisure Sciences, School of Health Sciences, University of KwaZulu-Natal, KwaZulu-Natal, South Africa; andrew.mckune@canberra.edu.au; 2 Department of Human Performance, College of Allied Health and Nursing, Minnesota State University, Mankato, MN 56001, USA; kristen.konkol@mnsu.edu; 3 Discipline of Sport and Exercise Science, UC-Research Institute for Sport and Exercise, Faculty of Health, University of Canberra, Bruce, ACT 2617, Australia

**Keywords:** physical activity, obesity, immunity, children, salivary biomarkers

## Abstract

This study examined whether cardiorespiratory fitness (CRF) and body composition are associated with salivary secretory immunoglobulin A (SIgA), a mucosal immunity marker, and salivary alpha-amylase (sAA), a marker of stress-related sympathetic nervous system (SNS) activity, in South African children. Morning (7:30–8:00 a.m.) saliva samples were collected from 132 children (10.05 ± 1.68 years old, 74 females, 58 males). Body composition, resting blood pressure, and predicted maximal aerobic capacity (VO_2max_) were determined, and SIgA and sAA were quantified. Obese children had significantly higher sAA compared with overweight and normal weight children (*p* < 0.01). SIgA secretion rate was significantly lower in obese and overweight vs. normal weight children (*p* < 0.01). Multiple-linear regression analysis revealed that body mass index (BMI) (*p* < 0.05) and diastolic blood pressure (DBP) (*p* < 0.05) were independent predictors of sAA with CRF acting as a mitigator. Age and BMI predicted SIgA secretion rate (*p* < 0.05) with BMI (*p* < 0.001) found to be an independent predictor of SIgA secretion rate. Obesity, based on BMI, was associated with elevated SNS activity and lowered mucosal immunity. CRF-mitigated sympathetic activation was not associated with mucosal immunity.

## 1. Introduction

Obesity and associated diseases, such as type 2 diabetes, have reached worldwide epidemic proportions [1]. In particular, childhood obesity is a focus of many public health efforts. In the United States alone, more than 17% of youth are obese [2] and statistics in South Africa reflect a similar problem [3,4,5]. Traditional and cultural perceptions regarding body size, urbanization, poor diet, low socioeconomic status, and lack of physical activity are a few of the suggested contributing factors [4,6].

The combination of obesity and lack of physical activity (PA) have been shown to have negative effects on immune and neuro-endocrine function [7,8]. Research examining the association between body composition, aerobic capacity, and immunity in children is still limited. However, from the studies available, it appears that both obesity and PA influence immunity. Cieslak, Frost, and Klentron [7] found that children who spent more time in sport activities had higher aerobic fitness and reported fewer “sick” days compared to those with body fat > 25%. Overweight children have been found to have double the risk of upper respiratory tract infections (URTI) compared with normal weight children, independent of PA levels [9]. Additionally, an increased risk of recurrent acute respiratory infections has been identified in preadolescent children with low PA levels when compared to those who were moderately or highly active [8,10]. Salivary secretory immunoglobulin A (SIgA) plays an important role in mucosal immunity. This antibody—which acts as the first line of defense against infection at mucosal surfaces by preventing colonization by microbes [8,11]—has been found to significantly correlate with reported URTIs [7]. Pallaro, Barbeito, et al. [12], found lower levels of total SIgA in obese compared with lean children, despite not observing an increase in the incidence of clinical symptoms and infections in the obese children. Past research in adults suggests that acute psychological stressors evoke transient increases in SIgA, whereas exposure to chronic stress leads to decreases in SIgA [13]. Links between long-term stress exposure and SIgA reduction have also been noted in children [14,15]. Thus, SIgA can serve as an important biomarker for examining the stress response in children.

There are a number of studies that have examined the role of the hypothalamic–pituitary adrenal axis in obesity, using salivary cortisol as a marker [15,16,17]. However, research on the other component of the stress system, the autonomic nervous system (ANS) is limited. An ideal way to study the functioning of the ANS of an individual, is by using salivary alpha-amylase (sAA) as a surrogate marker of stress-related ANS activity. Research has shown a possible link between sAA and body mass index (BMI). BMI has been found to be negatively associated with average morning sAA, with a 3.4% decrease in average sAA levels for each increasing point on the BMI scale [18]. Strahler, Rosenloecher, et al. examined sAA stress reactivity across different age groups and found that age and BMI were the strongest predictors of sAA increases, and that children had significantly higher baseline sAA activity than both groups of adult participants [19]. Links have also been identified between exercise and sAA. Response patterns of sAA to both physical and psychological stressors have been reported to correspond with response patterns of the sympathetic nervous system (SNS) [20]. However, most studies have focused on the adult population [21,22,23,24,25]. From the limited paediatric research available, it appears that exercise results in increases in sAA. Capranica, Lupo, et al. [26] observed peak sAA in young, male athletes at the end of a taekwondo competition, suggesting that these competitions may produce high stress in young athletes, the cause of which may be psychological or the result of the physical intensity of the competition and/or repeated performances.

Although the influence of obesity and PA on immunity has been well reported, research in the paediatric population is still limited, particularly in South Africa. The aim of this study was to investigate the effect that obesity has on both mucosal immunity and the SNS in black, South African children from a low socioeconomic area, and whether PA may mitigate these effects.

## 2. Methods

### 2.1. Participants

From an urban school in a low socioeconomic area in Pietermaritzburg, KwaZulu-Natal, 132 black, South African children (74 females, 58 males; age 10.05 ± 1.68 years) participated in the study. The study was approved by the University of KwaZulu-Natal's Biomedical Ethics Research Committee and complied with the World Medical Association Declaration of Helsinki [27] regarding ethical conduct of research involving human subjects. Written informed consent was obtained from the parents/guardians of the participating children and the study information was communicated to them. The guardians/parents completed a medical history form that included sections on infectious, immune, and salivary gland disorders. They were also trained in the salivary collection procedure and were provided with standardised instructions regarding brushing teeth and the intake of food and drink on the morning of the saliva collection [28].

### 2.2. Saliva Sample Collection

Samples were collected from each school grade on a separate morning over the course of a week, starting with grade 3 on Monday and ending with grade 7 on Friday. The children were requested to rest for 20 min upon arriving in the school hall at 7:00 a.m. and to complete with the researchers a short health questionnaire and interview (Wisconsin Upper Respiratory Symptom Survey (WURSS-44) [29] and the Physical Activity Readiness Questionnaire (PAR-Q) [30]), in order to assess symptoms of sickness (e.g., fever, flu, diarrhoea) and readiness for physical activity. None of the participants reported symptoms that suggested illness, and there were no reports of “bleeding gums” or “toothache”. Saliva samples were collected at the same time from all participants (between 7:30 and 8:00 a.m., ≥60 min post breakfast) [28]. The samples were collected via unstimulated passive drool over a time period of five minutes. The saliva collection protocol that was followed is described in Starzak, Konkol, McKune [31].

### 2.3. Analyses of Salivary Markers

Salivary IgA and sAA activity levels were determined in duplicate by enzyme-linked immunoassays using the SIgA and sAA ELISA kits (Salimetrics, State College, PA, USA). The coefficients of variation (CV) of all duplicate samples were less than 10%. The results were expressed as absolute concentration (µg/mL SIgA or U/mL sAA) and salivary secretion rate (SIgA µg/min or sAA U/min). Salivary IgA and sAA secretion rate were calculated by multiplying absolute SIgA and sAA concentration by saliva flow rate (mL·min^−1^). Saliva flow rate was calculated by dividing the total amount of saliva obtained in each sample (mL) by the time taken to produce the sample (4 min) [32].

### 2.4. Body Composition, Cardiovascular Measurements, and Cardio-Respiratory Fitness

The following measures were determined after the saliva collection by an ISAK Level 2 Anthropometrist. Stature was measured to the nearest 1 mm using a portable stadiometer (Nagata bw-1122h, Nagata Scale Co., Ltd, Yong Kang City, Tainan, Taiwan) and mass was measured to the nearest 0.1 kg using a calibrated electronic scale (Nagata bw-1122h, Nagata Scale Co., Ltd, Yong Kang City, Tainan, Taiwan). Seated resting heart rate, measured to the nearest 1 bpm, and resting blood pressure, measured to the nearest 1 mmHg, were recorded after a resting period of 10 min. Body fat percentage was determined using a 4 site skinfold method [33]. Triceps, biceps, suprailiac, and subscapular skinfolds were measured on the right hand side of the body using Harpenden^©^ (West Sussex, UK-Quality Measurement, Ltd.) skinfold callipers. Each site was measured twice to the nearest 1 mm and the mean value was recorded. Waist (narrowest part of the torso) and hip (level of maximum extension of the buttocks) circumference were measured to the nearest 1 mm with a tape measure and the waist-to-hip ratio was calculated. Cardiorespiratory fitness (CRF) was assessed using the 20 m multi-stage shuttle run test that predicts an individual’s maximal aerobic capacity (VO_2max_) [34]. This test has been shown to be an appropriate predictor of CRF for the age groups participating in the study [35].

### 2.5. Statistical Analyses

The data was tested for normality using the one-sample Kolmogorov–Smirnov test [36]. Descriptive statistics (mean ± SD) were calculated. One-way ANOVA with Tukey’s post hoc analysis were used to determine the differences in body composition, cardiovascular measures, CRF, and SIgA and sAA concentrations and secretion rates among the BMI categories. The BMI categories were determined using the growth charts published by the USA Center for Disease Control and Prevention (CDC) for BMI in boys and girls, 2–20 years of age. According to these CDC-BMI-for-age standards, the participants were grouped into the following CDC-BMI-for-age categories: normal weight (<85th percentile), overweight (≥85th percentile to <95th percentile), and obese (≥95^th^ percentile) [37].

In addition, the significance of associations between body composition, cardiovascular measures, CRF, and SIgA and sAA concentrations and secretion rates were determined using multiple linear regression (stepwise method) analyses. The concentration of SIgA and sAA and the secretion rate of SIgA and sAA were set as the dependent variables, and the body composition, cardiovascular, and CRF measures as the independent variables. All the independent variables were correlated against themselves and their R squared values were checked for multi-collinearity. Independent variables with R squared values >0.75 indicating high collinearity were removed from the regression analysis. Significance was set at *p* ≤ 0.05. All statistics were run using the IBM SPSS (version 19).

## 3. Results

Demographics, body composition, cardiovascular measures, CRF, and SIgA and sAA concentration and secretion rates were divided according to the three BMI categories (normal weight, overweight, and obese) based on the USA CDC growth charts and are presented as mean ± standard deviation in Table 1.

**Figure 1:**
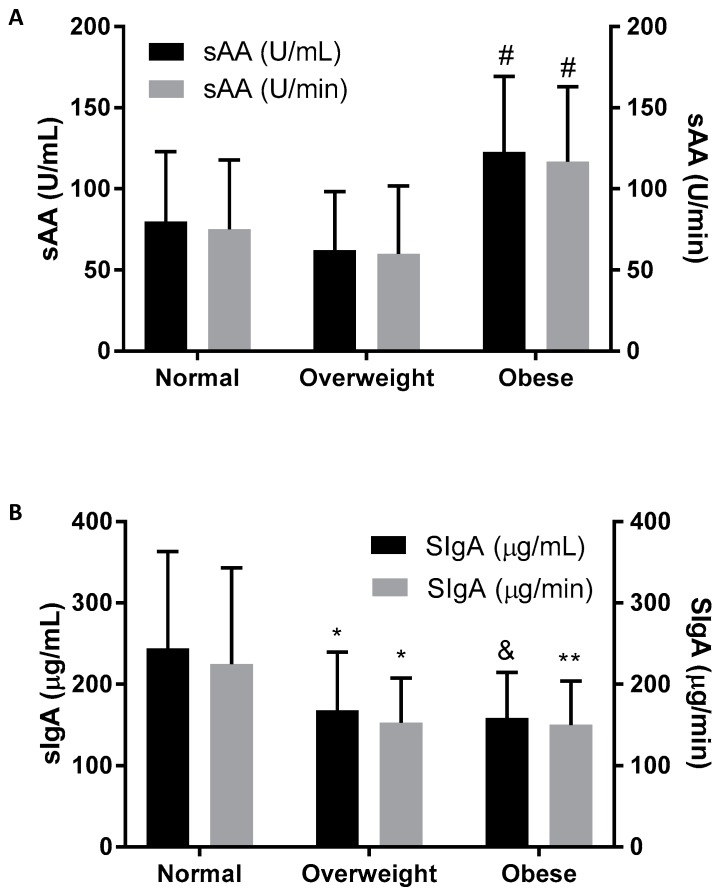
(A) sAA activity (U/mL) and sAA secretion rate (U/min), and (B) SIgA concentration (µg/mL) and SIgA secretion rate (µg/min), in normal, overweight and obese children. Values are mean ± standard deviation; * = p < 0.01 for overweight vs. normal weight; ** = p < 0.01 for obese vs. normal weight; & = p < 0.001 obese vs. normal weight, # = p < 0.001 for obese vs. normal weight and overweight.

The results of the one-way ANOVA examining the differences by BMI categories are indicated in Table 1 and Figure 1. There were significant differences in mass (F = 83.64, df = 2, 129, *p* < 0.0001), BMI (F = 193.36, df = 2, 129, *p* < 0.0001), waist-to-hip ratio (F = 193.36, df = 2, 129, *p* < 0.0001), body fat percentage (F = 336.98, df = 2, 129, *p* < 0.0005), systolic blood pressure (SBP) (F = 5.72, df = 2, 129, *p* < 0.005), diastolic blood pressure (DBP) (F = 291.76, df = 2, 129, *p* < 0.0001), VO_2max_ (F = 521.00, df = 2, 129, *p* < 0.0001), sAA activity (F = 17.05, df = 2, 129, *p* < 0.0001), sAA secretion rate (F = 15.15, df = 2, 129, *p* < 0.0001), SIgA concentration (F = 11.30, df = 2, 129, *p* < 0.0001), and SIgA secretion rate (F = 8.08, df = 2, 129, *p* < 0.001), among children of different BMI categories. Tukey’s post hoc analyses revealed that obese children had significantly (*p* < 0.01) higher mass, BMI, body fat percentage, DBP, SBP, and sAA activity and secretion rate, compared to overweight and normal weight children, as well as a significantly lower aerobic capacity (VO_2max_) than both normal (*p* < 0.001) weight and overweight (*p* < 0.05) children. In addition, SIgA concentration and secretion rate were significantly lower between normal weight and obese children (*p* < 0.01).

The multiple linear regression results are presented in Table 2. The model that best predicted sAA and accounted for 16.82% (R square = 0.1682, adjusted R square = 0.1487) of the variance was: sAA (U/mL) = 6.964 + 1.934 × [BMI] + 0.9208 × [DBP] − 0.7321 × [VO_2max_]. This model was significant (F = 8.63, *p* < 0.0001) and BMI (*p* < 0.05) and DBP (*p* < 0.05) were found to be independent predictors of sAA activity.

The model that best predicted sAA secretion rate and accounted for 14.84% (R square = −1682, adjusted R square = 0.1285) of the variance was sAA (U/min) = −5.314 + 2.351 × [BMI] + 0.7135 × [DBP] − 0.2394 × [VO_2max_]. This model was significant (F = 7.44, *p* < 0.0001) and BMI (*p* < 0.05) was found to be an independent predictor of sAA secretion rate.

The model that best predicted SIgA concentration and accounted for 13.86% (R square = 0.1386, adjusted R square = 0.1251) of the variance was SIgA (µg/mL) = 303.71 − 1.681 × [Age] − 45.737 × [BMI category]. This model was highly significant (F = 10.29, *p* < 0.0001) and BMI category (*p* < 0.05) was found to be an independent predictor of SIgA concentration.

The model that best predicted SIgA secretion rate and accounted for 9.32% (R square = 0.0932, adjusted R square = 0.0791) of the variance was SIgA (µg/min) = 233.04 + 2.210 × [Age] − 34.886 × [BMI × Category]. This model was highly significant (F = 6.58, *p* < 0.0019) and BMI category (*p* < 0.001) was found to be an independent predictor of SIgA secretion rate.

## 4. Discussion

Although the negative effects of obesity and lack of physical activity on immune and neuro-endocrine function have been identified in adults, information is limited for children [7,8,38]. This study investigated the effect that body composition and CRF have on salivary biomarkers of mucosal immunity as well as on SNS activation in the paediatric population, and is the first such study conducted on South African children from a low socioeconomic area. The main study findings revealed that BMI, DBP, and VO_2max_ predict sAA (with BMI and DBP as independent predictors and CRF as a mitigator) and that age and BMI category predict SIgA (with BMI as an independent predictor). This indicates that obesity (based on the BMI categories), as well as elevated DBP and poor CRF, play major roles in elevating resting SNS activation and lowering mucosal immunity.

The results demonstrated that obese children had significantly greater weight, BMI, body fat percentage, DBP, and SBP, compared to overweight and normal weight children. A number of other studies have also demonstrated that obese children display elevated blood pressure, BMI, and body fat percentage [39,40,41,42]. Our study also showed that obese children had a significantly lower aerobic capacity (VO_2max_) than both normal weight and overweight children. Similarly, Berndtsson, Mattsson, et al. [43] found lower relative VO_2max_ in Swedish obese children and adolescents when compared to the reference group.

A higher sAA activity and secretion rate were observed in obese children compared to normal weight or overweight children in our study. This finding is supported by Granger, Kivlighan, et al. [24] and Granger, Kivlighan, et al. [38] who found a positive association between sAA activity and increased body BMI (greater obesity) in adolescent males and females. However, Nater, Rohleder, et al. [18] found that BMI in the adult population was negatively associated with average morning sAA. Similarly, De Oliveira, Collares, et al. [44] found that obese boys presented with lower sAA activity compared to the control group (they did not note this for obese girls). Differences in the results relating to the association between BMI and sAA may be related to differences in the saliva collection protocol. In the study by De Oliveira, Collares, et al. [44], saliva was collected after chemical stimulation with lemon juice using a standardized technique. In the present study, saliva was collected via unstimulated passive drool which is considered to be the standard method for collection [45]. However, total sAA production was not reduced among obese boys, suggesting that the decreased enzyme concentration in obese boys may be compensated for by greater salivary secretion. Importantly, our results also indicate that DBP is related to sAA, and that increased aerobic fitness plays a role in reducing sAA. The SNS has a powerful effect on blood pressure, and an increased sympathetic drive has been recognised in patients with borderline hypertension [46]. Therefore, a reduction in sAA activity is beneficial for reducing the risk of developing hypertension. High levels of sAA have been shown to signify psychological stress in young adults, whereas a decrease in sAA is considered to be an indicator of stress reduction [47]. Thus, the relationship between aerobic fitness and sAA found in our study suggests that a high aerobic capacity may have a positive effect on the psychological wellbeing of children. Additionally, the increased sAA activity observed after exercise (as noted in other studies [48]) may serve to improve the protective effect of saliva since this enzyme is known to inhibit bacterial attachment to oral surfaces [25]. It is possible that this may be a compensatory response to the lower SIgA levels observed in the obese children. Short-term increases in sAA may be useful to the body as energy is made available by increased digestive action in response to stress [20]. Physiological stress reactions comprise orchestrated actions throughout the body, putting the organism in a state of overall preparedness to engage in fight or flight. Thus, increases in sAA activity may be one of many actions involved in activating the body’s resources to cope with stressful events or threats to homeostasis [20]. Further studies are needed to examine long-term changes in sAA activity.

Our study also demonstrated significantly lower SIgA concentration and secretion rate in obese compared with normal weight children, suggesting that mucosal immunity may be reduced in obese children. This finding is supported by Pallaroa, Tabernerb, et al. [48] who also observed lower levels of total SIgA in obese compared to lean children. Additionally, Jedrychowski, Maugeri, et al. [9] found that overweight children (BMI > 20) had double the risk of URTIs compared with normal weight children. Past research has indicated that exposure to long-term stress results in SIgA reduction in children [14,49]. Chronic stress has been shown to be associated with visceral obesity, type 2 diabetes, and related cardiometabolic complications [50]. On the other hand, obesity has been shown to promote systemic low-grade inflammation which can chronically stimulate the stress system [50,51,52]. Our results also indicated that there is a decrease in SIgA with increasing age, which is in line with past research [53,54]. SIgA levels are found to reach their approximate peak by 7 years of age, remain consistently high during mid-life, and then decline during old age [54].

It is important to recognise the study’s limitations. Although salivary flow rate was controlled and clear instructions were provided to the parents and participants regarding brushing teeth and dietary and hydration practices prior to saliva collection, the health condition of the children’s gums and teeth was not determined using standardised methods. Furthermore, only one saliva sample was taken per child because of logistical and financial constraints. Additionally, an examination for Tanner stages for sexual maturation [55] was not performed.

## 5. Conclusions

This study provides support for an association between poor CRF and/or obesity, and compromised mucosal immunity and SNS activation in children. Results show that obese children have elevated sAA, lowered SIgA, and poor CRF. Replication of the study with a larger sample size is required together with longitudinal follow up of clinical outcomes. This may contribute to a better understanding of the pathways mediating the enhancement of mucosal immunity, control of SNS activation, and chronic disease in children and, subsequently, in adults.

## Figures and Tables

**Table 1:** Demographic, body composition, cardiovascular and cardiorespiratory fitness data, and salivary alpha-amylase (sAA) and salivary secretory immunoglobulin A (SIgA) concentrations and secretion rates for normal weight, overweight, and obese children.

*n* = 132	Normal (*n* = 74) (males 36; females 38)	Overweight (*n* = 22) (males 8; females 14)	Obese (*n* = 36) (males 14; females 22)
Age (years)	10.26 (1.74)	9.59 (1.71)	9.97 (1.52)
Stature (cm)	140.25 (11.42)	141.20 (10.74)	143.53 (9.19)
Mass (kg)	34.10 (7.58)	40.83 (8.73) *	58.03 (11.85) ^#^
BMI ^bb^ (kg/m^2^)	17.11 (1.63)	20.20 (1.49) ***	28.03 (4.55) ^#^
Waist-Hip Ratio	0.77 (0.04)	0.79 (0.04) ^&&^	0.84 (0.07) ^&^
Body Fat %	19.56 (5.47)	28.18 (4.11) ***	39.38 (5.37) ^#^
Resting HR (b/min)	87.00 (12.57)	85.09 (11.21)	89.11 (12.01)
SBP (mmHg)	94.20 (12.20)	96.27 (9.67) ^&&^	107.28 (11.67) ^&^
DBP ^aaa^ (mmHg)	63.22 (8.33)	64.64 (8.17)	75.22 (10.00) ^#^
VO_2max_ (mL/kg/min)	29.35 (5.67)	25.97 (4.01) ^a^	22.34 (3.02) ^&,aa^
sAA (U/mL)	79.83 (43.12)	62.13 (36.06)	122.75 (46.50) ^#^
sAA (U/min)	75.07 (42.64)	59.91 (41.97)	116.75 (46.21) ^#^
SIgA (µg/mL)	243.95 (119.23)	167.92 (71.46) *	158.34 (56.03) ^&^
SIgA (µg/min)	224.88 (118.45)	152.95 (54.91) *	150.05 (53.98) **

Values are mean ± standard deviation; * = *p* < 0.01 for overweight vs. normal weight, ** = *p* < 0.01 for obese vs. normal weight, *** = *p* < 0.001 for overweight vs. normal weight, ^&^ = *p* < 0.001 obese vs. normal weight, ^&&^ = *p* < 0.01 for obese vs. overweight, ^#^ = *p* < 0.001 for obese vs. normal weight and overweight, ^a^ = *p* < 0.05 overweight vs. normal weight, ^aa^ = *p* < 0.05 obese vs. overweight, ^bb^ = body mass index, ^aaa^ = diastolic blood pressure.

**Table 2:** Multiple linear regression results for sAA activity, sAA secretion rate, SIgA concentration, and SIgA secretion rate.

Models		B *	Std. Error *	t *	*p* *
sAA (U/mL)	(Constant)	6.964	38.75	0.18	0.86
	BMI	1.934	0.92	2.09	0.04 *
	DBP	0.9208	0.46	2.00	0.04 *
	VO_2max_	−0.7321	0.76	0.96	0.34
sAA (U/min)	(Constant)	−5.314	39.24	0.14	0.89
	BMI	2.351	0.94	2.51	0.01 *
	DBP	0.7135	0.47	1.53	0.13
	VO_2max_	−0.2394	0.73	0.31	0.76
SIgA (µg/mL)	(Constant)	303.71	58.28	5.21	<0.0001
	Age	−1.681	5.27	0.32	0.75
	BMI Category (1 = normal, 2 = overweight, 3 = obese)	−45.737	10.09	4.54	<0.0001 *
SIgA (µg/min)	(Constant)	233.04	56.97	4.09	<0.0001
	Age	2.210	5.15	0.43	0.67
	BMI Category (1 = normal, 2 = overweight, 3 = obese)	−34.886	9.86	3.54	0.0006 *

* Coefficients.

## References

[1] Alwan A. (2011). Global Status Report on Noncommunicable Diseases 2010.

[2] Ogden C.L., Carroll M.D., Kit B.K., Flegal K.M. (2014). Prevalence of childhood and adult obesity in the United States, 2011–2012. JAMA.

[3] Goedecke J., Jennings C., Lambert E. (2006). Obesity in South Africa. Chronic Diseases of Lifestyle in South Africa Since 1995–2005.

[4] Steyn K. (2006). Conceptual framework for chronic diseases of lifestyle in South Africa. Chronic Diseases of Lifestyle in South Africa: 1995–2005.

[5] Reddy S.P., Resnicow K., James S., Funani I.N., Kambaran N.S., Omardien R.G., Masuka P., Sewpaul R., Vaughan R.D., Mbewu A. (2012). Rapid increases in overweight and obesity among South African adolescents: Comparison of data from the South African National Youth Risk Behaviour Survey in 2002 and 2008. Am. J. Public Health.

[6] Puoane T., Steyn K., Bradshaw D., Laubscher R., Fourie J., Lambert V. (2002). Obesity in South Africa: The South African demographic and health survey. Obes. Res..

[7] Cieslak T., Frost G., Klentron P. (2003). Effect of physical activity, body fat and salivary cortisol on mucosal immunity in children. J. Appl. Physiol..

[8] Walsh N.P., Gleeson M., Shephard R.J., Gleeson M., Woods J.A., Bishop N.C., Fleshner M., Green C., Pedersen B.K., Hoffman-Goetz L. (2011). Position statement part one: Immune function and exercise. Exerc. Immunol. Rev..

[9] Jedrychowski W., Maugeri U., Flak E., Mroz E., Bianchi I. (1998). Predisposition to acute respiratory infections among overweight preadolescent children: An epidemiologic study in Poland. Public Health.

[10] Jedrychowski W., Maugeri U., Flak E., Mroz E., Bianchi I. (2001). Cohort study on low physical activity level and recurrent acute respiratory infections in schoolchildren. Cent. Eur. J. Public Health.

[11] Teeuw W., Bosch J.A., Veerman E.C., Nieuw Amerongen A.V. (2004). Neuroendocrine regulation of salivary IgA synthesis and secretion: Implications for oral health. Biol. Chem..

[12] Pallaro A., Barbeito S., Taberner P., Marino P., Franchello A., Strasnoy I., Ramos O., Slobodianik N. (2002). Total salivary IgA, serum C3c and IgA in obese school children. J. Nutr. Biochem..

[13] Segerstrom S.C., Miller G.E. (2004). Psychological stress and the human immune system: A meta-analytic study of 30 years of inquiry. Psychol. Bull..

[14] Shirtcliff E.A., Coe C.L., Pollak S.D. (2009). Early childhood stress is associated with elevated antibody levels to herpes simplex virus type 1. Proc. Natl. Acad. Sci. USA.

[15] Wallerius S., Rosmond R., Ljung T., Holm G., Björntorp P. (2003). Rise in morning saliva cortisol is associated with abdominal obesity in men: A preliminary report. J. Endocrinol. Investig..

[16] Björntorp P., Rosmond R. (2000). Obesity and cortisol. Nutrition.

[17] Champaneri S., Xu X., Carnethon M.R., Bertoni A.G., Seeman T., DeSantis A.S., Diez Roux A., Shrager S., Golden S.H. (2013). Diurnal salivary cortisol is associated with body mass index and waist circumference: The multiethnic study of atherosclerosis. Obesity.

[18] Nater U.M., Rohleder N., Scholotz W., Ehlert U., Kirschbaum C. (2007). Determinants of the diurnal couse of salivary alpha-amylase. Psychoneuroendrocrinology.

[19] Strahler J., Mueller A., Rosenloecher F., Kirschbaum C., Rohleder N. (2010). Salivary a-amylase stress reactivity across different age groups. Psychophysiology.

[20] Nater U., Rohleder N. (2009). Salivary alpha-amylase as a non-invasive biomarker for the sympathetic nervous system: Current state of research. Psychoneuroendocrinology.

[21] Chatterton R., Vogelsong K., Lu Y., Ellman A., Hudgens G. (1996). Salivary alpha-amylase as a measure of endogenous adrenergic activity. Clin. Physiol..

[22] Ljungberg G., Ericson T., Ekblom B., Birkhed D. (1997). Saliva and marathon running. Scand. J. Med. Sci. Sports.

[23] Steerenberg P., van Asperen I., van Nieuw Amerongen A., Biewenga J., Mol D., Medema G. (1997). Salivary levels of immunoglobulin A in triathletes. Eur. J. Oral. Sci..

[24] Granger D., Kivlighan K., Blair C., El-Sheikh M., Mize J., Lisonbee J.A., Buckhalt J.A., Stroud L.R., Handwerger K., Handwerger K. (2006). Integrating the measurement of salivary alpha amylase into studies of child health, development, and social relationships. J. Soc. Pers. Relat..

[25] Walsh N., Blannin A., Clark A., Cook L., Robson P., Gleeson M. (1999). The effects of high-intensity intermittent exercise on saliva IgA, total protein and alpha-amylase. J. Sports Sci..

[26] Capranica L., Lupo C., Cortis C., Chiodo S., Cibelli G., Tessitore A. (2012). Salivary cortisol and alpha-amylase reactivity to taekwondo competition in children. Eur. J. Appl. Physiol..

[27] World Medical Association (2013). World medical association declaration of helsinki: ethical principles for medical research involving human subjects. JAMA.

[28] Salimetrics Improving Saliva Study Results. https://www.salimetrics.com/newsletter-v1/november-2010.

[29] Barrett B., Locken K., Maberry R., Schwamman J., Brown R., Bobula J., Stauffacher E.A. (2002). The Wisconsin upper respiratory symptom survey (WURSS). J. Fam. Pract..

[30] Thomas S., Reading J., Shephard R.J. (1992). Revision of the physical activity readiness questionnaire (PAR-Q). Can. J. Sport Sci..

[31] Starzak D.E., Konkol K.F., McKune A.J. (2016). Twelve weeks of soccer-specific training: Effects on mucosal immunity, salivary alpha-amylase and body composition in male African youths. Sport Sci. Health.

[32] Novas A., Rowbottom D., Jenkins D. (2003). Tennis, incidence of URTI and Salivary IgA. Int. J. Sports Med..

[33] Brook C. (1971). Determination of body composition of children from skinfold measurements. Arch. Dis. Child..

[34] Ledger L., Lambert J. (1982). A maximal multistage 20 m shuttle run test to predict VO_2max_. Eur. J. Appl. Physiol..

[35] Tomkinson G.R., Leger L.A., Olds T.S., Cazorla G. (2003). Secular trends in the performance of children and adolescents (1980–2000): An analysis of 55 studies of the 20 m shuttle run test in 11 countries. Sports Med..

[36] Daniel W.W. (1990). Applied Nonparametric Statistics.

[37] Ogden C.L., Flegal K.M. (2010). Changes in terminology for childhood overweight and obesity. Natl. Health Stat. Rep..

[38] Granger D., Kivlighan K., El-Sheikh M., Gordis E., Stroud L. (2007). Salivary alpha-amylase in biobehavioral research. Ann. N. Y. Acad. Sci..

[39] He Q., Ding Z.Y., Fong D.Y.-T., Karlberg J. (2000). Blood pressure is associated with body mass index in both normal and obese children. Hypertension.

[40] Figueroa-Colon R., Franklin F.A., Lee J.Y., Aldridge R., Alexander L. (1997). Prevalence of obesity with increased blood pressure in elementary school-aged children. South. Med. J..

[41] Farpour-Lambert N.J., Aggoun Y., Marchand L.M., Martin X.E., Herrmann F.R., Beghetti M. (2009). Physical activity reduces systemic blood pressure and improves early markers of atherosclerosis in pre-pubertal obese children. J. Am. Coll. Cardiol..

[42] Lurbe E., Alvarez V., Liao Y. (1998). The impact of obesity and body fat distribution on ambulatory blood pressure in children and adolescents. Am. J. Hypertens..

[43] Berndtsson G., Mattsson E., Marcus C., Larsson U.E. (2007). Age and gender differences in VO_2max_ in Swedish obese children and adolescents. Acta Paediatr..

[44] De Oliveira C., Collares E., Barbieri M., Fernandes M. (1996). Production and concentration of saliva and salivary amylase in obese children. Arq. Gastroenterol..

[45] Beltzer E.K., Fortunato C.K., Guaderrama M.M., Peckins M.K., Garramone B.M., Granger D.A. (2010). Salivary flow and alpha-amylase: Collection technique, duration, and oral fluid type. Physiol. Behav..

[46] Julius S. (1991). Autonomic nervous system dysregulation in human hypertension. Am. J. Cardiol..

[47] Takai N., Yamaguchi M., Aragaki T., Eto K., Uchihashi K., Nishikawa Y. (2004). Effect of psychological stress on the salivary cortisol and amylase levels in healthy young adults. Arch. Oral. Biol..

[48] Pallaroa A., Barbeitob S., Tabernerb P., Marinob P., Franchellob A., Strasnoyb I. (2002). Total salivary IgA, serum C3c and IgA in obese school children. J. Nutr. Biochem..

[49] Vermeer H.J., van IJzendoorn M.H., Groeneveld M.G., Granger D.A. (2012). Downregulation of the immune system in low-quality child care: The case of secretory immunoglobulin A (SIgA) in toddlers. Physiol. Behav..

[50] Kyrou I., Tsigos C. (2009). Stress hormones: Physiological stress and regulation of metabolism. Curr. Opin. Pharmacol..

[51] Mathieu P., Lemieux I., Després J.P. (2010). Obesity, inflammation, and cardiovascular risk. Clin. Pharmacol. Ther..

[52] Rocha V.Z., Libby P. (2009). Obesity, inflammation, and atherosclerosis. Nat. Rev. Cardiol..

[53] Miletic I., Schiffman S., Miletic V., Sattely-Miller E. (1996). Salivary IgA secretion rate in young and elderly persons. Physiol. Behav..

[54] Kugler J., Hess M., Haake D. (1992). Secretion of salivary immunoglobulin A in relation to age, saliva flow, mood states, secretion of albumin, cortisol, and catecholamines in saliva. J. Clin. Immunol..

[55] Tanner J.M. (1962). Growth at Adolescence: With a General Consideration of the Effects of Hereditary and Environmental Factors upon Growth and Maturation from Birth to Maturity.

